# A Comparison of the Treatment Outcomes With and Without the Use of Intra-articular Corticosteroids for Frozen Shoulder Manipulation

**DOI:** 10.7759/cureus.44427

**Published:** 2023-08-31

**Authors:** Kemal Kayaokay, Derya Arslan Yurtlu

**Affiliations:** 1 Department of Orthopaedics and Traumatology, Izmir Katip Çelebi University, Izmir, TUR; 2 Department of Anaesthesiology and Reanimation, Izmir Katip Çelebi University, Ataturk Training and Research Hospital, Izmir, TUR

**Keywords:** intra-articular corticosteroid injection, frozen shoulder, adhesive capsulitis, physiotherapy rehabilitation, manipulation

## Abstract

Background

Manipulation under anesthesia is known to be an effective treatment method for a frozen shoulder. However, this process is painful and causes difficulty in early physiotherapy. Intra-articular corticosteroids may relieve pain after manipulation. This study compared patients who underwent manipulation under anesthesia with those who only underwent physiotherapy and those who received intra-articular corticosteroid administration and physiotherapy.

Methodology

A total of 33 patients presenting with frozen shoulders were included in this study. Those who underwent manipulation after anesthesia were determined as group 1 (16 patients) and those who received intra-articular corticosteroids in addition to manipulation under anesthesia were determined as group 2 (17 patients). Pain was evaluated using the Visual Analog Scale (VAS) scores. Functional outcomes were assessed using the University of California-Los Angeles (UCLA) scores and shoulder range of motion (ROM).

Results

VAS and UCLA scores of both groups were similar at 12 weeks and six months. ROM improved significantly after manipulation in both group 1 and group 2 (p < 0.05). There was no significant difference between the ROM in the two groups after manipulation and physiotherapy. Only the external rotation ROM value was better in group 2 (p = 0.032)

Conclusions

Physiotherapy after manipulation is a successful treatment method for frozen shoulder patients. It reduces pain in the early period compared to patients who are not administered intra-articular corticosteroids. However, it has no functional superiority.

## Introduction

A frozen shoulder causes severe limitation of active and passive range of motion (ROM) of the glenohumeral joint and a decrease in the activities of daily living due to severe pain [1]. The incidence of frozen shoulder has been reported at 2-5% [2]. Its incidence increases over the age of 50 [3]. It was also named adhesive capsulitis by Neviaser because of chronic inflammation in the joint capsule, increase in capsule thickness, and progressive fibrosis in the joint capsule shown on the pathological examination of the disease [4]. It is an inflammatory process that affects all soft tissues such as the joint capsule, subacromial scholarship, rotator interval, axillary recess, and biceps tendon sheath. The etiopathogenesis of the disease has not been fully elucidated. However, diseases such as diabetes mellitus, hyperlipidemia, thyroid metabolism disorders, and hypoadrenalism, which often accompany the clinical picture, are thought to play a role in its etiopathology [5]. Although the etiopathogenesis of frozen shoulder is controversial, the clinical picture of the disease is quite typical and restrictive. Cases where the underlying pathology is thought to be due to diseases such as diabetes mellitus, hyperlipidemia, thyroid metabolism disorders, and hypoadrenalism are called secondary frozen shoulder [6-8]. Frozen shoulder is a disease that is usually manipulated under anesthesia and physical therapy should be started on the same day. However, the first days of physical therapy may be painful and movements may be limited. This study aimed to investigate the effect of intra-articular corticosteroid administration on pain, joint ROM, and success of physiotherapy at an early stage after manipulation.

## Materials and methods

This study was conducted among patients who presented to the Orthopaedics and Traumatology Clinic with a frozen shoulder after obtaining the approval of the İzmir Kâtip Çelebi University Non-interventional Clinical Research Ethics Committee (approval number: 0199-2023). Clinical and laboratory data of patients hospitalized for frozen shoulder between January 2017 and December 2022 were retrospectively reviewed. Patients older than 18 years of age, who underwent standard manipulation procedures under deep sedation in the operating room, who started physiotherapy on the same day after manipulation, and who regularly performed physiotherapy for 12 weeks were included in the study. Patients with a rotator cuff tear in the shoulder, who previously had a shoulder-related pathology, who underwent open arthroscopic surgery, and who had a history of fractures or trauma to the same limb were excluded from the study.

Standard manipulation under anesthesia in the chair position was performed as arm abduction, flexion, external rotation when the arm was in neutral position, and external and internal rotation when the arm was in 90° abduction [9]. The preference for intra-articular corticosteroid administration was the practitioner’s own choice and was made at random. Generally, practitioners preferred long-acting betamethasone dipropionate 6.43 mg (equivalent to 5 mg betamethasone) for intra-articular injections. It was also mixed with 1% lidocaine hydrochloride as a local anesthetic. In this study, the same standard manipulation was applied to all patients by different surgeons. After the manipulation, physiotherapy was started on the same day in the service, accompanied by a physiotherapist. Patients who underwent physiotherapy and regularly attended the first-week, sixth-week, and 12th-week follow-ups were included in the study. Retrospectively, patients were divided into the following two groups: patients who underwent manipulation only (group 1), and patients who received intra-articular corticosteroids after manipulation (group 2).

Demographic characteristics of the patients, including age, gender, and comorbidities, were recorded. Preoperative and postoperative ROM (flexion-abduction-external rotation-internal rotation) were measured using a goniometer. The University of California-Los Angeles (UCLA) shoulder scores were compared functionally at the pre-manipulation and post-manipulation controls [10]. Pain was recorded using the Visual Analog Scale (0: no pain; 10: the worst pain ever experienced) [11].

SPSS Statistics version 22 (IBM Corp., Armonk, NY, USA) software was used for data analysis. For comparisons between the groups, the Pearson chi-square test and Fisher’s exact test were used to evaluate categorical data, and the Mann-Whitney U-test was used for statistical analysis of continuous data that did not correspond to normal data. P-values <0.05 were considered statistically significant.

## Results

A total of 39 patients presented to our clinic between 2017 and 2022 and were diagnosed with a frozen shoulder. Of these, 33 patients who were over the age of 18 and could be followed up for 12 weeks were included in this study. Overall, 11 of the patients participating in the study were male and 22 were female. The mean age of the 33 patients in our study was 53.03 ± 10.25 years. There were 16 patients in group 1 and 17 patients in group 2. While the mean age of the patients was 52.69 ± 8.49 years in group 1, which underwent manipulation only, it was 53.35 ± 11.93 years in group 2, which underwent manipulation and intra-articular injection. Frozen shoulder was more common in women aged 40-60 years.

The mean ROM of patients in group 1 before manipulation was 86.25° for flexion, 79.06° for abduction, 39.69° for external rotation, and 31.25° for internal rotation. In group 2, the mean ROM before manipulation was 87.94° for flexion, 81.76° for abduction, 39.71° for external rotation, and 34.41° for internal rotation. The ROM in group 1 ROM after manipulation was 123.13° for flexion, 111.56° for abduction, 71.25° for external rotation, and 68.75° for internal rotation. In group 2, it was measured as 133.53° for flexion, 118.24° for abduction, 82.35° for external rotation, and 78.82° for internal rotation. Group 1 external rotation change ROM difference was significantly higher than group 2 (Table 1).

**Table 1:** Comparison of ROM preoperatively and postoperatively. ROM: range of motion; SD: standard deviation

ROM	Group 1		Group 2	
	Preoperative	Postoperative	Preoperative	Postoperative
Flexion (mean ± SD)	86.25 ± 12.22	123.13 ± 21.97	87.94 ± 6.62	133.53 ± 12.59
Abduction (mean ± SD)	79.06 ± 12.93	111.56 ± 16.60	81.76 ± 7.2	118.24 ± 12.61
External rotation (mean ± SD)	39.69 ± 12.31	71.25 ± 17.17	39.71 ± 10.52	82.35 ± 10.77
Internal rotation (mean ± SD)	31.25 ± 12.17	68.75 ± 17.07	34.41 ± 7.68	78.82 ± 11.92

ROM increased significantly after manipulation in both group 1 and group 2 (p < 0.05). There was no significant difference between the ROM of the two groups after manipulation and physiotherapy. After manipulation, there was a statistically significant difference in external rotation ROM in group 2 compared to group 1, but no difference was observed between other ROMs (Table 2).

**Table 2:** Comparison of ROM 12 weeks after treatment. ROM: range of motion; SD: standard deviation

ROM	Group	Group 2	P-value
Flexion	123.13 ± 21.97	133.53 ± 12.59	0.103
Abduction	111.56 ± 16.60	118.24 ± 12.61	0.202
External rotation	71.25 ± 17.17	82.35 ± 10.77	0.032
Internal rotatıon	68.75 ± 17.07	78.82 ± 11.92	0.057

The VAS and UCLA scores before and after manipulation were similar in both groups (Table 3).

**Table 3:** Comparison of VAS and UCLA scores of both groups. VAS: Visual Analog Scale; UCLA: University of California-Los Angeles; SD: standard deviation

	Group 1		Group 2		P-value
	Preoperative	Postoperative	Preoperative	Postoperative	
VAS (mean ± SD)	7.44 ± 1.31	1.37 ± 1.25	6.59 ± 1.27	0.76 ± 1.03	0.137
UCLA (mean ± SD)	10.38 ± 5.03	29.56 ± 4.38	12.76 ± 4.45	31.76 ± 3.36	0.114

## Discussion

The results of the study showed that intra-articular corticosteroid administration after manipulation was not superior to the control group except for external rotation movements.

In frozen shoulder, the goal is to achieve a painless ROM of the joint. Achieving this goal includes a wide spectrum of treatments such as manipulation, intra-articular injection, hydration, ultrasonography-guided injections, platelet-rich plasma (PRP) injection, conservative therapy, and arthroscopic loosening [12,13]. While the issue of which treatment is superior has been the subject of debate, it has led to many studies comparing these options. In general, conservative treatment, analgesia, and physiotherapy are performed. In patients who do not experience improvement and an increase in movements for more than three to six months, manipulation or arthroscopic relaxation is performed under anesthesia.

The goal of applying corticosteroids after manipulation treatment is to suppress new inflammation that will occur after manipulation as well as to reduce pain [14,15]. Reducing pain is important in increasing the success of physiotherapy in the early period [16]. In this study, pain was evaluated with VAS and there was no difference between the two groups. Kiwimaki et al. compared manipulation and isolated physiotherapy programs and found that there was no significant difference between them at the end of the one-year follow-up [17]. Afzal et al. and Mobini et al. reported that corticosteroid along with physiotherapy was more successful than physiotherapy alone [18,19]. Ranalletta et al. found that patients who received corticosteroid injections along with physiotherapy experienced a faster reduction in pain and improved shoulder motion and function [20]. Khallaf et al. reported that the functional results of patients who received intra-articular corticosteroids were more successful after physiotherapy [21]. Song et al. reported that hydrodistension under manipulation was more successful than corticosteroid injection [22]. Shahzad et al., in their study of 202 patients, reported improvement in VAS score, UCLA, and ROM after intra-articular injection of PRP compared to intra-articular injection of corticosteroids [23]. Ryans et al. reported significant improvement in patients receiving intra-articular corticosteroids at week six in a randomized controlled trial [24]. They also reported that physiotherapy improved external rotation at six weeks. In our study, external rotation was significantly better in group 2 patients at 12 weeks. We attributed this to our injection from the posterior shoulder. Therefore, it led us to predict that intra-articular corticosteroid administration from different regions can increase other movements.

Gam et al. reported that 18 patients had good ROM after 15 years of follow-up [25]. In our study, there was no difference between the sixth and 12th-week follow-up of patients who received intra-articular corticosteroid injection after manipulation and those who did not. Again, the sixth-month follow-up was similar. However, in the first days after manipulation, the pain was less in patients who received corticosteroid injections in terms of VAS score. UCLA scores were similar in both groups after manipulation. Compared to before the manipulation, both groups showed improvement in UCLA scores.

Physiotherapy after manipulation is a successful treatment modality in frozen shoulder patients. There are studies that report functional improvement after intra-articular corticosteroids are added to physiotherapy. Oh et al. reported that physiotherapy performed after an intra-articular injection of a single dose of corticosteroid led to better functional outcomes than physiotherapy alone [26]. However, intra-articular corticosteroid administration reduces pain in the early period.

The limitation of our study was that it was retrospective and had a small patient group. Another limitation is that patients were not classified according to their daily performance differences. The short follow-up period is also one of the limitations of this study.

## Conclusions

Manipulation and physiotherapy under anesthesia is a successful treatment modality in the treatment of frozen shoulder. In our study, intra-articular corticosteroid administration after manipulation increased external rotation ROM. Intra-articular corticosteroid administration after manipulation has no superiority in terms of pain and functional scores.
